# Complete Remission of Methotrexate-Related Epstein-Barr-Virus-Associated Hodgkin-Like Lymphoma following Withdrawal of MTX Coupled with Clarithromycin Administration

**DOI:** 10.1155/2012/658745

**Published:** 2012-12-19

**Authors:** Nobuo Takemori, Hiroyuki Kaneko, Masaya Nakamura, Masaru Kojima

**Affiliations:** ^1^Division of Hematology, Department of Internal Medicine, Imai Hospital, Tanaka-cho 100, Ashikaga, Tochigi 326-0822, Japan; ^2^Department of Orthopedics, Imai Hospital, Tanaka-cho 100, Ashikaga, Tochigi 326-0822, Japan; ^3^Department of Surgery, Imai Hospital, Tanaka-cho 100, Ashikaga, Tochigi 326-0822, Japan; ^4^Department of Anatomic and Diagnostic Pathology, School of Medicine, Dokkyo Medical University, Mibu-machi, Shimotsuga-gun, Tochigi 321-0293, Japan

## Abstract

Patients with rheumatoid arthritis (RA) are known to develop lymphoproliferative disorders (LPDs) during the course of illness, particularly in cases treated with methotrexate (MTX) for long periods. We describe a case of MTX-related Epstein-Barr-virus-(EBV-) associated LPD resembling Hodgkin's lymphoma (HL), in which a dramatic complete remission was achieved after withdrawal of MTX coupled with clarithromycin (CAM) administration. Withdrawal of MTX coupled with CAM administration seemed to be effective for treating MTX-related EBV-associated LPDs. In particular, an immunomodulative effect of CAM might have been involved in achieving complete remission.

## 1. Introduction

Rheumatoid arthritis (RA) patients have an increased risk of developing lymphoma [1–3]. Since Ellman's first report [4] on lymphoma in a patient with RA who received low-dose methotrexate (MTX), the relationship between MTX medication and development of lymphoproliferative disorders (LPDs) has been widely discussed [5–8]. In 1993, Kamel et al. [9] reported that two patients taking MTX for rheumatologic disease, who developed lymphoma, were associated with Epstein-Barr virus (EBV) infection, and a spontaneous regression occurred after withdrawal of MTX. Later, numerous reports on MTX-related EBV-associated lymphomas, which showed regression or remission after withdrawal of MTX, have been published [10–15].

Clarithromycin (CAM), a member of macrolide family, is a widely used antibacterial drug. It is also known to have other important pharmacological effects such as immunosuppression or immunomodulation [16]. In this paper, we report a case of MTX-related EBV-associated Hodgkin-like lymphoma, the so-called Hodgkin-like lesion [17, 18], in which a complete remission was achieved after withdrawal of MTX coupled with CAM administration. A possible immunomodulatory effect of CAM in the treatment of MTX-related EBV-associated LPD is emphasized with special reference to circulating cytokine levels. 

## 2. Case Report

**Figure 1:**
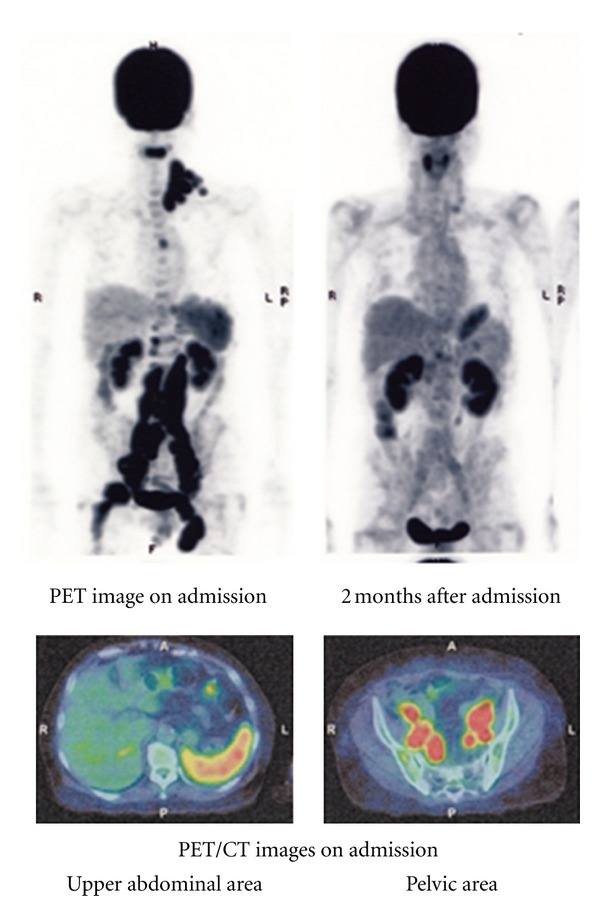
Imaging test results: the coronal PET image (upper left) shows wide spread pathological uptake of radiotracer (FDG) in cervical, supraclavicular, mediastinal, para-aortic, peri-iliac and inguinal lymph nodes and spleen. A second PET/CT scan performed 2 months after admission (upper right) shows complete resolution of the abnormal FDG activity. Fused PET/CT image on admission (lower left) shows moderate FDG uptake in the spleen.

A 60-year-old woman (body weight: 51 kg) with a 10-year history of seropositive RA was admitted to Imai Hospital on march 12, 2011, because of an intermittent high fever, for 3 months, early satiety, anorexia, weight loss, joint pains, and left cervical swelling. For approximately 10 years before initial presentation, she had been treated with several medications including MTX, salazosulfapyridine, bucillamine, prednisolone, and nonsteroidal anti-inflammatory agents. In particular, MTX treatment (at weekly doses of 2 mg then gradually increased to 8 mg) was started approximately 6 years before admission. Physical examination on admission showed left cervical, supraclavicular, and inguinal lymph node swellings. The metacarpal and pharyngeal joints of both hands and bilateral knee and ankle joints were slightly swelled with minimal tenderness. A complete blood count showed anemia (red blood cell count, 3.34 × 10^12^ /L; hemoglobin 9.4 g/dL), leukocytopenia (white blood cell count, 1.3 × 10^9^ /L with 83% neutrophils, 17% lymphocytes), and normal platelet count (155 × 10^9^ /L). Elevated levels of CRP (7.43 mg/dL; nl. range 0~0.26 mg/dL), LDH (299 IU/L; nl. range 106–211 IU/L), soluble-IL-2 receptors (s-IL-2R) (3,080 IU/mL; nl. range 124~466 IU/mL), *β*2-microglobulin (3.4 mg/L; nl. range 0.9~1.9 mg/L), RAPA (×160; nl. range <×40) and ferritin (1,720 ng/mL; nl. range 39.4~340 ng/mL), and reduced levels of serum Fe (18 *μ*g/dL; nl. range 54~181 *μ*g/dL) and albumin (2.2 g/dL; nl. range 3.9~4.9 g/dL) were confirmed. Titers of EB virus antibodies were elevated in VCA-IgG (×320; nl. range <×10), and EBNA (×40; nl. range <×10) with VCA-IgM level less than ×10. Slightly elevated levels of IL-1*β* (2.99 pg/mL; nl. range ≦0.928 pg/mL), IL-6 (4.99 pg/mL; nl. range ≦2.41 pg/mL) and TNF-*α* (3.09 pg/mL; nl. range ≦1.79 pg/mL) were observed in the serum obtained 2 weeks after admission. No increments of serum IL-2, IL-10, and IL-12 were seen. A positron emission computed tomography (PET/CT) scan showed notable swelling of cervical, supraclavicular, mediastinal, para-aortic, peri-iliac, and inguinal lymph nodes with splenic involvement (Figure 1). 

**Figure 2:**
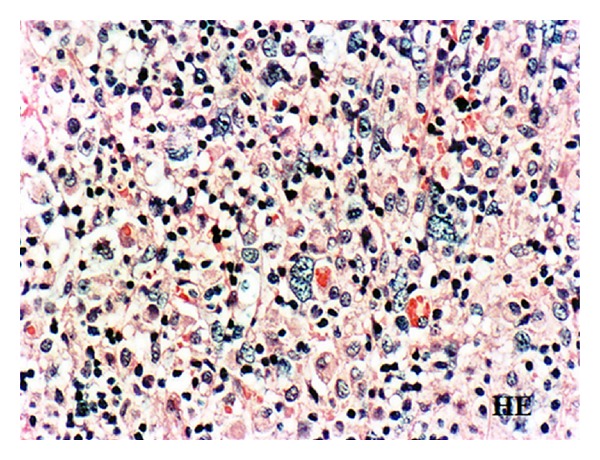
Histology of the cervical lymph node biopsy showing the infiltration of giant cells reminiscent of Reed-Sternberg cells and atypical large round cells with remarkable nucleoli in the background of lymphocytes, original magnification ×300. H&E stain.

**Figure 3:**
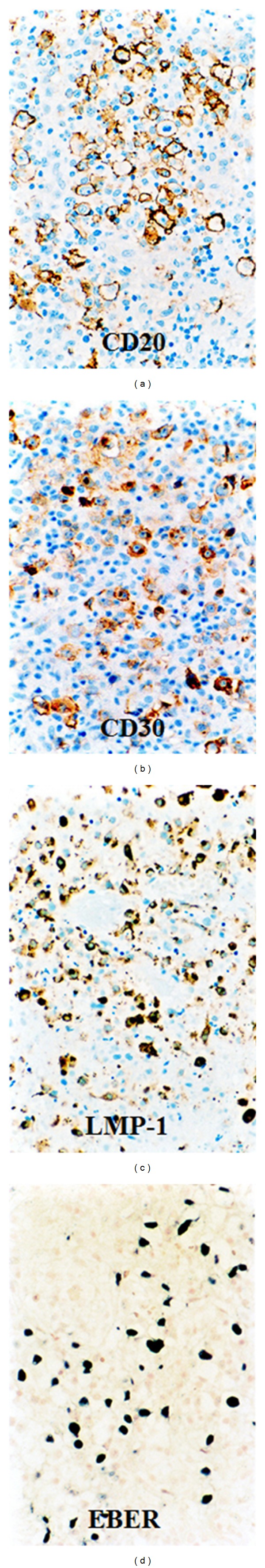
Immunohistochemical stainings for CD20, CD30 and LMP-1 and EBER staining (in situ hybridization). Tumor cells are positive for CD20, CD30, LMP-1 and EBER, original magnification ×300.

A left cervical lymph node biopsy showed the infiltration of giant cells reminiscent of Reed-Sternberg cells and atypical large round cells with remarkable nucleoli in the background of small lymphocytes (Figure 2). Follicular structures were absent. The giant cells and large round cells were immunohistochemically positive for CD20, CD30, and LMP-1, but negative for CD15. In situ hybridization also revealed the presence of EBV-encoded small RNA (EBER) in these cells (Figure 3). Thus, a diagnosis of MTX-related EBV-associated B-cell LPD, the so-called Hodgkin-like lesion, was made. However, neither immunoglobulin heavy chain gene (JH) nor T-cell receptor chain gene (TCR-C*β*1) was demonstrated by Southern blot hybridization analysis of the lymph node. 

In this case, all antirheumatic medicines were suspended and an oral administration of CAM (100 mg at 12-hour intervals) was initiated on the day of admission. After the initiation of this treatment, the fever disappeared within one week. The elevated serum value of CRP returned to normal within 2 weeks, and the elevated s-IL-2R level returned to normal within 2 months. The follow-up PET/CT scan 2 months after admission showed complete disappearance of the FDG uptake, which had been demonstrated in the first PET/CT scan. The serum IL-1, IL-6, and TNF-*α* levels returned to normal after achieving complete remission. Although the complete remission was rapidly achieved, RA symptoms including the joint pain and swelling gradually reappeared. Thus, administration of prednisolone (10 mg/day) was restarted 5 months after admission. CAM was discontinued one year after its initiation. No unfavorable adverse effects were observed with CAM. The patient currently remained in complete remission with stable RA controlled by prednisolone as of September, 2012. 

## 3. Discussion

RA patients have an increased risk of developing hematopoietic and other malignancies. The epidemiologic studies have shown that the relative risk of developing lymphoma in patients with RA or rheumatologic diseases is estimated to be 2.0 to 5.5 times higher than the general population [2, 3, 19]. Although the pathogenesis of RA-associated lymphomas is still unclear, the increased risk appears to be related to the high inflammatory activity of rheumatologic disease, immunosuppressive agents such as MTX for RA or EBV infection [20]. MTX is regarded as an effective immunosuppressive agent for the treatment of autoimmune diseases, especially RA [21], but its long-term use can lead to severe immunodeficiency during the course of illness. The fact that spontaneous remissions or regressions occur in MTX-related LPDs after withdrawal of MTX highlights a likely causative role of this drug in the development of LPDs [11]. Mariette et al. [7] reported that, despite the risk of non-Hodgkin's lymphoma (NHL) was not significantly increased in RA patients treated with MTX, their incidence of classical Hodgkin's lymphoma appeared to be higher. Nevertheless, the attribution of MTX to LPD is not thought to be significant because the reported incidence in RA patients is low [11]. LPDs are known to develop occasionally in individuals with immunodeficiencies. According to the recent World Health organization (WHO) classification for lymphoid neoplasms [22], immunosuppressive conditions prior to LPDs are categorized into primary immune disorders; human immunodeficiency virus infection; iatrogenic immunosuppression in patients receiving solid organ or bone marrow allografts, that is, posttransplant PTLDs; and other iatrogenic immunodeficiency-associated LPDs. It is noteworthy that approximately 50% of MTX-LPDs are EBV-positive [6]. According to Hoshida et al. [23], the EBV-positive rate in PTLDs was described to be 63–95%, whereas that in sporadic LPDs in Western countries was only 5%. Considering the significant difference of EBV positivity between LPDs with immunodeficiency and sporadic ones, the severity of immunodeficiency seems to be associated with EBV infection or reactivation. EBV is known to be associated with several malignancies, including Burkitt's lymphoma, undifferentiated nasopharyngeal carcinoma, Hodgkin's lymphoma, nasal T-cell lymphoma, gastric carcinoma, and B-cell lymphoma in immunocompromised individuals. Irrespective of its causes, the immunodeficiency itself is presumed to provide a basis for the development of some malignant lymphomas, probably through the activation of EBV [23]. Particularly, patients with RA have a T-lymphocyte defect that allows EBV-infected B-lymphocytes to survive [11]. Miyazaki et al. [24] reported that EBV-positive lymphoma in RA has a strong tendency to undergo a spontaneous remission after the withdrawal of MTX. However, it is also true that there are some EBV-negative lymphomas which regressed after MTX withdrawal [6, 7]. Thus, EBV-positivity is not necessarily related to spontaneous regression or remission.

 Macrolides are well known as antibiotics but also have other important pharmacological effects such as immunosuppression or immunomodulation [16]. Cytokines and chemokines are known to be key regulators of the inflammatory response, with both pro-inflammatory (e.g., TNF-*α*, granulocyte-macrophage colony-stimulating factor, IL-1, IL-6, IL-8, and interferon-*γ*[IFN-*γ*]) and anti-inflammatory (e.g., IL-10) effects [25]. Generally, macrolides are known to decrease the production of pro-inflammatory cytokines that are detrimental to the host. Hamada et al. [26] demonstrated that erythromycin (macrolide analogous to CAM) treatment increased the serum levels of IFN-*γ* and IL-4, causing cytotoxic macrophages to increase their potency in killing tumor cells. In clinical field, it was reported that long-term treatment with CAM (400 mg/day) following basic anticancer therapy (chemotherapy and/or radiotherapy) significantly increased the median survival time of patients with advanced non-small-cell lung cancer [27]. 

Majima et al. [28] demonstrated that CAM treatment enhanced IL-12 mRNA andIFN-*γ* mRNA and suppressed the production of IL-10 mRNA by peripheral blood mononuclear cells obtained from unresectable non-small lung cancer patients. IL-12 is known to regulate the production of IFN-*γ* by activating NK cells, consequently enhancing antitumor activity [29, 30]. Majima et al. [31] demonstrated that administration of CAM induced the expression of IL-12 mRNA in the spleen cells obtained from Lewis lung carcinoma-bearing mice. They also assumed that the production of IL-12 during CAM treatment, which was observed in both human and mice, could lead to activation of NK cells [32] and production of IFN-*γ* by Th1 cells [28], resulting in tumor regression. In addition, Sassa et al. [33] reported that CAM treatment inhibited the expression of TGF-*β*, TNF-*α*, and matrix methalloproteinase-9 in a model of transplanted tumors in rats. Sakamoto et al. [34] demonstrated that the long-term CAM treatment for inoperable non-small lung cancer patients suppressed the production of cachexia-related cytokines such as IL-6 and TNF-*α*. IL-6 is a multifunctional cytokine that regulate the immune response, hematopoiesis, acute phase response, and inflammation [35]. IL-6 is known to activate STAT3 and thereby activates proliferative and antiapoptotic pathways [36]. Recently, Hinrichs et al. [37] analyzed the plasma levels of IL-6 in 38 PTLDs undergoing treatment (chemotherapy) for PTLD and demonstrated that pretherapeutic levels of IL-6 were elevated and their levels were correlated with the course of disease; falling in responders and rising in nonresponders. Sugiyama et al. [38] evaluated the effect of macrolides on T-cell regulation by murine bone-marrow-derived dendritic cells (DCs); CAM significantly upregulated the expression of CD80 (costimulatory molecule for T-cell activation) in DCs and inhibited the production of IL-6 by DCs. Moreover, CAM significantly decreased IL-2 production when naive splenic T-cells were cocultured with DCs stimulated with lipopolysaccharides and exposed to CAM. Recently, Xu et al. [39] demonstrated in the mouse experiment that bone marrow stromal cells induced apoptosis of lymphoma cells in the presence of IFN-*γ*, and TNF, and the apoptotic effect was mediated by nitric oxide. Hamada et al. [40] experimentally demonstrated by using Lewis lung carcinoma-inoculated mice that delayed initiation of CAM treatment resulted in the enhancement of natural killer cell activity and CD8+ T-cell cytotoxicity and increased the number of IFN-*γ* producing T-cells and IL-4 producing T-cells. Gu et al. [41] examined the circulating levels of cytokines in B-cell NHL (B-NHL), and reported that increased s-IL-2R and decreased IL-13 were significantly associated with the risk of B-NHL, and TNF-*α*, s-TNF-*α* and IL-5 were marginally associated with risk of B-NHL. However, no association was observed between B-NHL risk and levels of other cytokines such as IL-1R, IL-2, IL-4, IL-6, IL-10, IL-12, IL-12p70, CRP, and s-TNF-R1. IL-10 is known as an immunosuppressive cytokine; it inhibits T-cell proliferation and production of IFN-*γ* and IL-2 by Th1 cells [42]. Majima et al. [28] reported that CAM treatment decreased IL-10 mRNA in peripheral blood mononuclear cells obtained from unresectable non-small lung cancer. In the present case, elevated serum levels of IL-1, IL-6 and TNF-*α* were confirmed after two weeks of CAM treatment, and their levels returned to normal after achieving complete remission. This finding might indicate that these cytokines are involved in developing lymphoma. Since the immunomodulatory mechanism of CAM seems to be far more complicated than expected, it is difficult to evaluate to what degree these cytokines are involved in developing lymphomas. Possibly, NK cells/cytotoxic T-cells and/or cytotoxic macrophages regained their normal or activated function after MTX withdrawal coupled with CAM treatment. Based on experimental and clinical studies, CAM seems to play an important role in reconstructing a well-balanced cytokine network. However, in the present case, CAM was administered simultaneously with the withdrawal of antirheumatic drugs. The possibility that the complete remission in the present case might have been achieved without CAM administration cannot be completely ruled out. Since neither immunoglobulin heavy chain gene (JH gene) nor TCR-C*β*_1_ gene was demonstrated, indicating a polyclonal pattern of proliferation, our case might have been at the initial stage of LPD, and this resulted in rapid complete remission. 

As for the treatment, if the clinical situation permits, a period of observation for spontaneous remission after withdrawal of MTX is recommended [6, 8, 11, 15]. Nevertheless, careful observation is required because the recurrence is common and may be treated successfully using standard chemotherapy [15]. The present case seems to indicate that if it was found to be polyclonal in proliferation, careful observation with CAM treatment as well as withdrawal of MTX may be recommended. If the lymphoma is found to be monoclonal in proliferation, the treatment including chemotherapy or radiotherapy is recommended without hesitation. Thus, genetic analysis of lymphoma cells is necessary to make a good choice of treatment. 

Concerning the optimal dose of CAM, Mikasa et al. [27] reported that a dose of 400 mg/day of CAM can be used as an adjuvant drug for cancer therapy with no remarkable adverse effects. In the present case, we administered the same dose for one year without troubles. It is interesting to note that CAM has recently been incorporated into the lenalidomide/dexamethasone regimen (BiRD) for treating multiple myeloma and a higher complete remission response has been obtained by this regimen [43].

In conclusion, the present case suggested helpful clues in the treatment of MTX-related EBV-associated LPDs occurred in RA patients. CAM seems to have important pharmacological effects such as immunosuppression or immunomodulation as well as an anti-infective role. In particular, it may be a promising adjuvant drug in treating a certain type of LPDs similar to the present case.
